# Antimicrobial Resistance: We Must Pursue a Collaborative, Global Approach and Use a “One Health” Approach

**DOI:** 10.3390/antibiotics8040237

**Published:** 2019-11-27

**Authors:** Dagan O Lonsdale, Jeffrey Lipman

**Affiliations:** 1 Department of Intensive Care Medicine, St George’s University Hospitals NHS Foundation Trust, London SW17 0QT, UK; daganlonsdale@googlemail.com; 2 Department of Clinical Pharmacology and Therapeutics, St George’s, University of London, London SW17 0RE, UK; 3 Royal Brisbane and Womens’ Hospital, University of Queensland, Brisbane 4029, Australia; 4 Nimes University Hospital, University of Montpellier, 30029 Nimes, France

Treating infection is a key part of the work of most clinicians. Whilst new drug technologies like biologics have begun a revolution in the treatment of cancer and autoimmune disease, there has been a conspicuous absence of new classes of antibiotic over the last 30 years. This, coupled with the mass use of antibiotics in farming and the continued emergence of resistant pathogens has created a perfect storm, and antimicrobial resistance is now viewed as a global public health emergency [1,2]. Combating the threat posed by the failure of current antibiotics presents a unique need to co-ordinate research and intervention policy across the spectrum of primary and secondary care, the private and public sector, and public health alongside working with colleagues in agriculture and farming aiming towards a “one health” approach. In this issue of *Antibiotics*, a variety of articles are presented that cover the breadth of human research in this field from in vitro work on novel therapies to commentary on public health strategies.

The emerging crisis of antibiotic resistance and paucity of novel therapies, has led to a resurrection of historic drug development pipelines. Robertson and Musiol-Kroll [3] provide a comprehensive account of the part actinomycetes have played in the history of antimicrobial therapy. The origins of ß-lactams, macrolides, and tetracyclines (among others) lie in the exploration of these organisms in the mid twentieth century and the article details their discovery and utility, as well as outlining the potential discovery pipeline for future development of naturally occurring antimicrobials. Previously discarded treatment options are also undergoing a resurgence. In their articles, Romero-Calle and colleagues [4] and Fernandez et al. [5] discuss the potential of bacteriophages (bactericidal viruses) in comprehensive summaries that discuss the history, mechanism of action, and current state of early phase research of these therapies. Mgbeahurulike et al. [6] utilize another strategy for developing new antimicrobial treatments by combining a novel synergistic compound with an established antibiotic. They provide evidence in their in vitro work of the synergistic effect of the alkaloids piperine and piplartin with rifampicin against *Staphylococcus aureus*. Infection caused by carbapenem-resistant Enterobacteriaceae (CRE) provide a particular challenge to clinicians worldwide. Suay-Garcia and Pérez-Gracia [7] provide a concise summary of the history, epidemiology and resistance mechanisms of these pathogens, and outline the treatment strategies that may be employed to treat them. Old (fosfomycin), newer (‘double carbapenem’), and novel (ceftazidime/avibactam) treatment strategies are described, with a clear message that global cooperation is paramount to combating CRE.

Antimicrobial stewardship, including the prevention of inappropriate antibiotic prescribing is key to preventing the continued rise and spread of resistant pathogens. However, there is currently no international consensus on the definition or accurate quantification of the global burden of inappropriate prescribing. Hood and colleagues [8] provide commentary on some of the audit tools available in Australia [9] and the USA [10] and present a novel approach, developed through an expert Delphi process, that they aim to use in UK secondary care. Al-Hasan et al. [11] in their review, argue for a more straightforward metric for antimicrobial stewardship performance–institutional antimicrobial use. In primary care, Colliers et al. [12] present an analysis of the burden of infection and antibiotic prescribing in out of hours contact between practitioners and patients in Belgium. They found that more than one in five out of hours appointments resulted in an antibiotic prescription. They also found that out of hours prescribing was often not in keeping with local guidelines. Sunde, Nygaard, and Høye [13] present some of the challenges faced by General Practitioners when deciding whether to give antimicrobial prescriptions, highlighting in their qualitative and quantitative study that patient expectations remain a significant driver of prescribing for practitioners. Grigoryan and colleagues [14] present a qualitative analysis of antimicrobial prescribing for perhaps one of the more common indications in primary care, urinary tract infections. They include a report on a wide variety of resources used by practitioners when making prescribing decisions, pointing out that stewardship interventions must consider where and how practitioners seek information. Borek et al. [15] further describe some of the barriers to success of antimicrobial stewardship interventions. They suggest some strategies, sourced from a stakeholder engagement exercise, to improve the success rates.

For many clinicians, the threat or challenge of managing infection due to antimicrobial resistant organisms is often focused on a single patient, ward or practice. In this issue, Holmes and Hughes discuss the wider health economic implications of failing to act to combat resistant pathogens [16]. The headline healthcare cost of no action, $100 trillion by 2050 [2], should prompt action from even the most skeptical of policymakers. However, the authors provide insightful commentary on the challenges in economic evaluation of interventions that may provide benefit to only individual patients or to populations over a long time period. They argue succinctly that economic assessment must be paired alongside evaluation of clinical efficacy of healthcare interventions to combat antimicrobial resistance, if funds are to be targeted efficiently and effectively. More broadly, it is clear that antimicrobial resistance is not an issue that is related to, or originates solely from humans. Although common sense dictates that policy and interventions to combat antimicrobial resistance must be multi-faceted and include stakeholders from public health, hospitals, and the community alongside colleagues from agriculture, farming and veterinary medicine. Singh et al. [17] provide a commentary of the situation in Singapore, pointing out that even in an economy with significant resource, combating antimicrobial resistance is complex and challenging to coordinate. Their work, based on a qualitative analysis from stakeholder interviews, highlights the need to understand and address cultural, social, and behavioral expectations of antibiotic use, alongside implementing public health policy.

Articles on in vitro work by Vianna et al. [18] on antimicrobial efflux pumps and Costa et al. [19] outlining the antimicrobial activity of silver camphro-imine complexes alongside work from Gavilán et al. [20] on a novel and sensitive assay for detecting low levels of antibiotic in animal feed, complete this innovative and exciting multi-disciplinary issue of *Antibiotics*.
